# A Case of Fibular Aplasia-Tibial Campomelia-Oligosyndactyly (FATCO) Syndrome Associated With Split Hand/Foot Syndrome With Long Bone Deficiency (SHFLD) and Review of the Literature

**DOI:** 10.7759/cureus.65162

**Published:** 2024-07-22

**Authors:** Theodora- Eleftheria Deftereou, Vaya R Karapepera, Christina Angelika Alexiadi, Stylianos Tologkos, Vasiliki Papadatou, Georgios Alexiadis, Dimitrios Karamanidis, Lambropoulou Maria

**Affiliations:** 1 Laboratory of Histology-Embryology, Democritus University of Thrace, Alexandroupolis, GRC; 2 ENT Clinic, General Hospital of Ioannina "G. Hatzikosta", Ioannnina, GRC; 3 Department of Radiology, Private Radiodiagnostic Center, Alexandroupolis, GRC; 4 Department of Obstetrics and Gynecology, University General Hospital of Alexandroupolis, Alexandroupolis, GRC

**Keywords:** tibial campomelia, fibular aplasia, ectrodactyly, shfld, fatco

## Abstract

Split hand/foot malformation is a heterogeneous congenital disorder mainly presented with a median cleft of hands or/and feet. It can be associated with long bone aplasia, a syndrome also known as split hand/foot syndrome with long bone deficiency (SHFLD), which is a very rare condition. We report a very rare case of a male fetus with SHFLD syndrome combined with fibular aplasia, tibial campomelia, and oligosyndactyly (FATCO) syndrome. FATCO syndrome is also an extremely infrequent congenital limb defect by itself. Based on our review of the literature, there appears to be no other FATCO case reported in Greece.

## Introduction

Congenital limb anomalies are common skeletal birth defects, affecting one in 1,000 births [1], with anomalies in upper limbs clearly dominating [2]. It has been estimated that over 15% of all limb defects concern the split-hand foot malformation (SHFM), a congenital distal limb absence mainly observed in the central rays of hands and/or foot [3,4].

In the literature, SHFM has been also described as “ectrodactyly”, “cleft hand”, “lobster claw hand”, “crab claw hand” and “symbrachydactyly” [5]. The incidence of SHFM is one in 18,000 newborns. Among those, 80% present with only a single abnormal limb, mainly affecting the upper limbs [6,7]. SHFM demonstrates a wide heterogeneity in clinical severity, even among different limbs of an individual, and generally is shown in an asymmetric pattern between left and right limbs [8].

The typical type is defined by the absence of the central rays resulting in a cleft appearance, but also oligodactyly, monodactyly, syndactyly, camptodactyly, clinodactyly, metacarpal or metatarsal, and phalangeal aplasia can occur [3,4,9]. This rare condition can be non-syndromic, mostly associated with autosomal dominant inheritance with variable expressivity and reduced penetrance [8], or part of a syndrome involving other extra-limb abnormalities [9], as more than 50 syndromes have been associated with SHFM [10]. The most frequent SHFM-associated syndromes are ectrodactyly-ectodermal dysplasia-cleft lip and palate (EEC), acro-derma-to-ungual-lacrimal-tooth (ADULT), lacrimo-auriculo-dento-digital (LADD), and limb-mammary syndrome [3,4].

SHFM associated with long bone deficiency has been termed the split hand/foot syndrome with long bone deficiency (SHFLD) syndrome, an extremely rare defect occurring in one in 1,000,000 newborns [11]. In this condition, SHFM is almost always accompanied by tibial aplasia or hypoplasia and generally an intact fibula. Less frequent long bone anomalies concern defects of the femur and ulnae [10,12,13].

On the other hand, fibular aplasia-tibial campomelia-oligosyndactyly (FATCO) syndrome was first described by Hetch et al. in 1981 [14]. Since then, only a few cases have been reported in the literature as fibular aplasia, tibial campomelia, and oligosyndactyly [15].

In this study, we report the postnatal findings in a fetus 21+6 weeks after elective termination of pregnancy due to multiple limb anomalies and we summarize the literature concerning these uncommon conditions.

## Case presentation

A 36-year-old healthy Caucasian primigravida with no exposure to radiation or drug intake, requested a pregnancy termination at 22 weeks after the second-trimester scan, as the fetus was diagnosed with upper and lower limb abnormalities. The parents were nonconsanguineous and family history was referred unremarkable. The fetus was delivered at the Laboratory of Histology-Embryology of Democritus University of Thrace (DUTH), Greece, and clinical examination and X-ray were performed to establish the diagnosis. 

**Figure 1:**
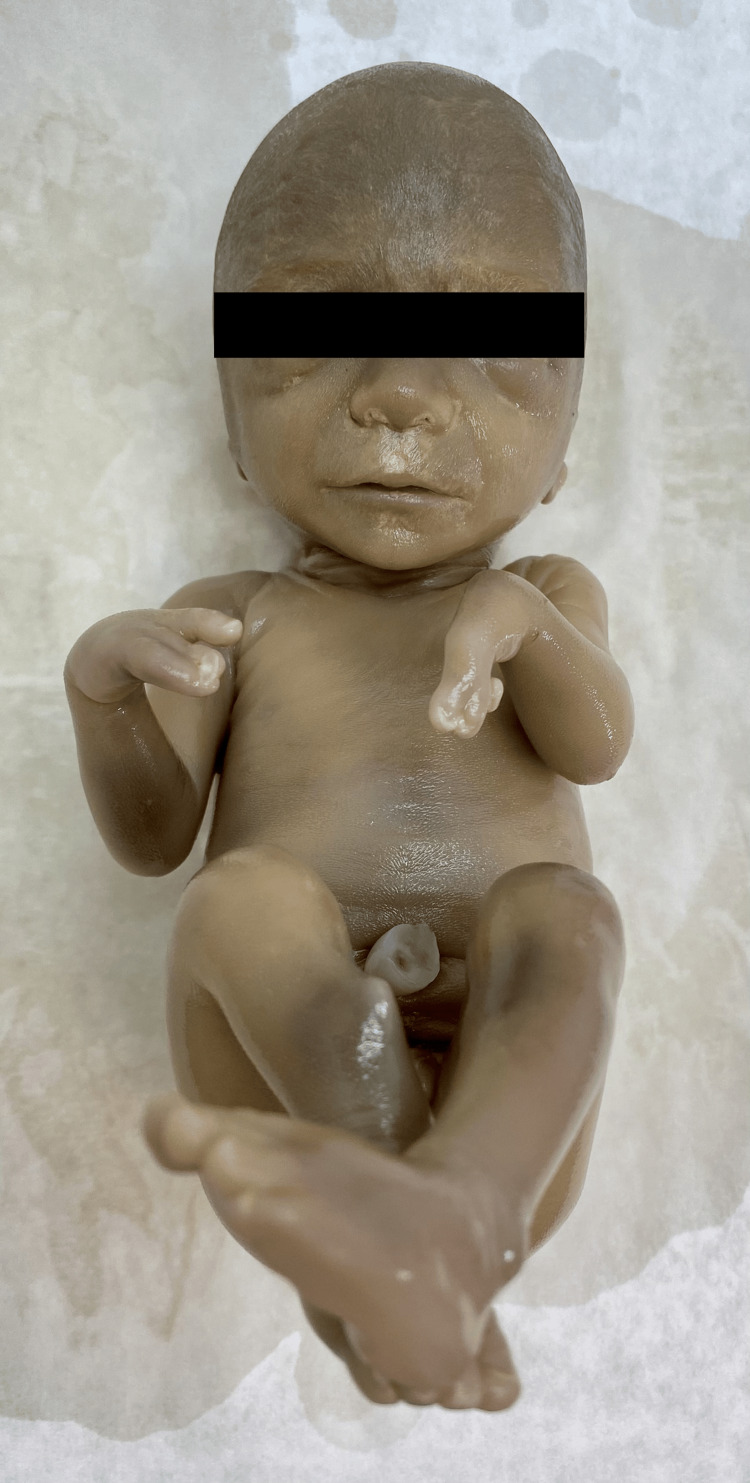
Post-abortum photograph of fetus showing bilateral ectrodactyly of upper limbs with right lower limb shortening, anterior bowing, and overlying skin dimpling with associated bilateral feet oligosyndactyly.

Fetal autopsy in upper limbs revealed bilateral split hand malformation with an asymmetric pattern in left-right abnormalities. Absence of both thumbs and bilateral oligosyndactyly were observed. Ectrodactyly of the right hand involved the absence of two digits (thumb and fifth) resulting in a median cleft and soft tissue syndactyly of second and third digits. On the left hand, the thumb and a central digit were missed, whereas the fifth digit was present but dysmorphic and hypoplastic (Figure 1). Clinical examination of the lower limbs showed shortening of the right limb, anterior bowing in the right tibia with skin dimpling, bilateral oligodactyly, and bilateral cutaneous incomplete syndactyly (Figure 1).

**Figure 2:**
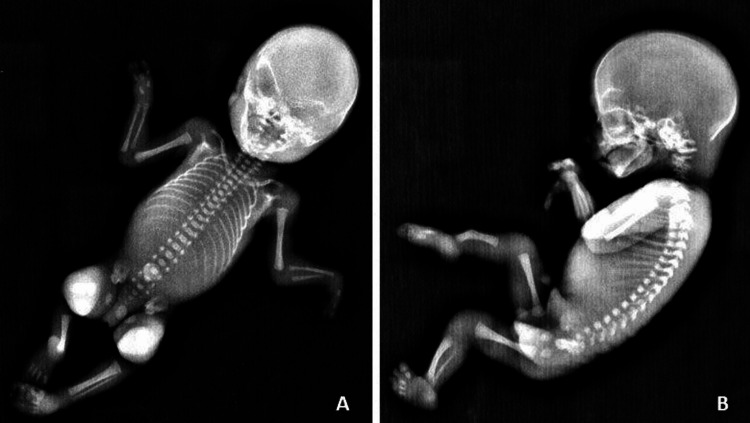
(A) Fetal X-ray showing bilateral hand malformations and left ulnae hypoplasia; (B) Radiograph of lower limbs showing right fibular aplasia and tibial campomelia with missing metatarsal bones and digits bilaterally.

Radiographic evaluation of upper limbs revealed missing metacarpal bones and digits, bilaterally. Ulnae hypoplasia was detected on the left limb. On the right hand, two metacarpals and three proximal and three distal phalanges were observed. The left hand comprised two metacarpals and two phalanges (Figure 2A). X-ray examination of lower limbs showed skeletal malformation on the right side with complete fibular aplasia. The presence of campomelic and shortened right tibia was also confirmed. On each foot, four metatarsal bones and four well-formed digits were observed (Figure 2B). 

No other anomalies of the spine, pelvis, or skull were detected. External genitalia were normal. No facial malformations or other external abnormalities were detected. Karyotype analysis revealed a normal male karyotype (46, XY).

## Discussion

In this report, we presented a case of a male fetus with both FATCO and SHFLD syndromes present. To the best of our knowledge, till date, this is the first report of a case with the coexistence of these two extremely rare syndromes in the literature.

**Figure 3:**
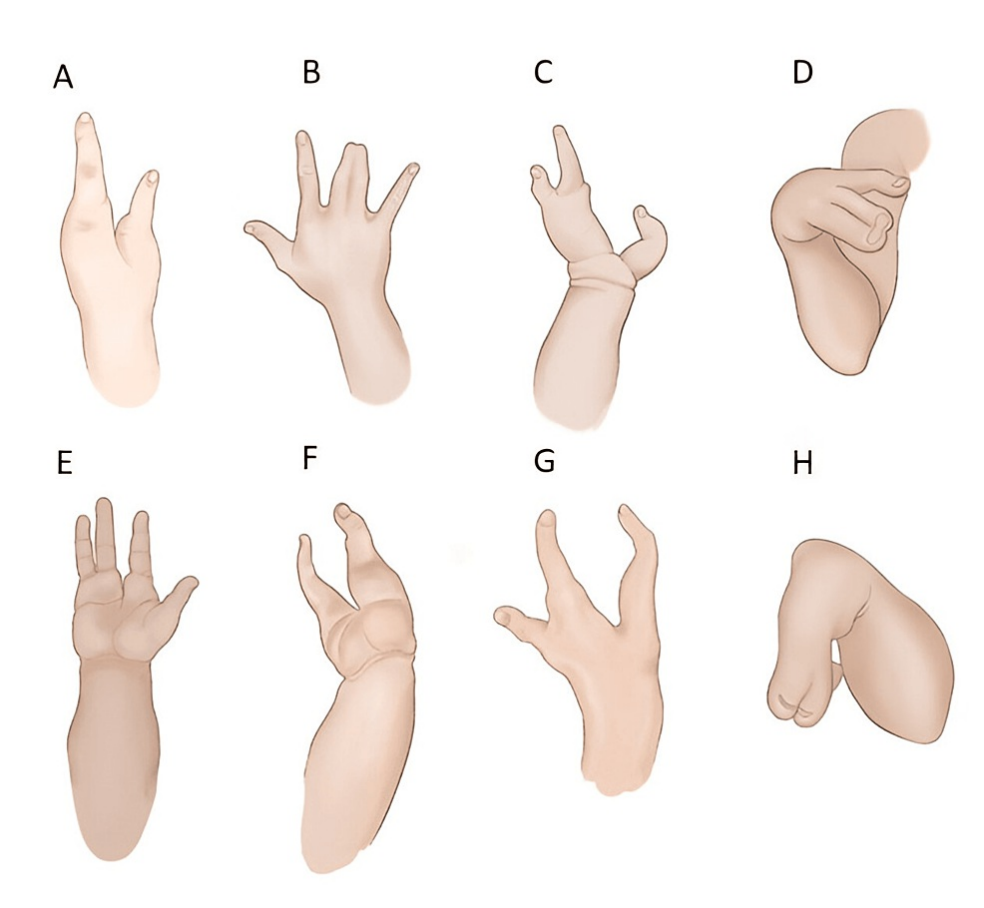
Schematic representation of various split hand malformations, including our case. (A) Dorsal view of bidactylous with thumb and one digit separating by a cleft; (B) Dorsal view of hand with syndactyly and two mild clefts; (C) Dorsal view of split hand with three malformed digits and large cleft extended to the carpometacarpal joint; (D) Absence of the thumb and one digit, median cleft, and syndactyly (right hand of our case); (E) Palmar view of a hand with the absence of the third digit and median cleft; (F) Dorsal view of ectrodactyly with only two digits present separating by a large cleft; (G) Dorsal view of split hand with thumb and two digits; (H) Ectrodactyly with thumb and a central digit absent, hypoplasia of fifth digit, and syndactyly of two central digits (left hand of our case). Image Credit: Vaya Karapepera

SHFLD is defined by the presence of upper or lower limb ectrodactyly and long bone defects. As previously reported, SHFLD is mainly associated with tibial aplasia or hypoplasia [12,16] and ectrodactyly phenotype may show significant heterogeneity, as presented in Figure 3.

SHFLD cases with defective femur, ulnae, or radius have also been reported [13,17,18]. To the best of our knowledge, no reported SHFLD case with tibial campomelia has been recorded in the literature [9,16,19]. Furthermore, a common locus for SHFLD is that it rarely affects the fibula [12]. These findings played a major role in leading us to strongly believe that in this case, two separate clinical syndromes coexist.

The most common long bone agenesis is considered to be fibular aplasia [20], which can occur besides FATCO syndrome, in other conditions like femur-fibula-ulna (FFU) syndrome, Furhmann syndrome, Du Pan syndrome, and thrombocytopenia-absent radius (TAR) syndrome [1]. The main diagnostic finding that can differentiate FATCO syndrome from other fibula-related entities is the absence of femoral and radial defects [21]. In particular, Furhmann syndrome which has been already presented in the literature to overlap with FATCO syndrome [22], in this case, was ab initio excluded by the confirmation of the straight femora. Normal femora should also rule out the diagnosis of FFU syndrome [1]. Fibular aplasia with ectrodactyly has been considered by some as the clinical entity that should include FATCO syndrome [23], but in the literature, there is no agreement [22,12]. Isik et al. recently suggested that ectrodactyly with fibular aplasia and no additional findings should be considered as FATCO syndrome [24]. However, as the etiology of FATCO remains unclear, the vast majority of cases demonstrating the clinical triad of fibular aplasia, tibial campomelia, and oligosyndactyly are considered FATCO syndrome [1,11,22,24].

It is suggested that the diagnosis of FATCO syndrome should initially be based on abnormalities of lower limbs. Then, all associated syndromes should be ruled out based on phenotypic features. Of course, the scenario of overlapping conditions should be kept in mind, as these clinical entities may share a common genetic basis [25].

In general, FATCO syndrome is an extremely rare condition, with less than 30 cases reported worldwide [1,2,26]. After a thorough review of the literature [1,2,11,12,14,15,20-48], we found that the total number of cases increases to 46 if FATCO variants are also included (Table 1) [27-29]. It is noteworthy to clarify that previous studies included some cases demonstrating the characteristics of FATCO syndrome which, however, had been diagnosed as fibular hemimelia [30,31]. On the other hand, two cases of Fuhrmann syndrome were presented by Huber et al. in 2003. Regarding these cases, the authors strongly believe that they should be counted as FATCO syndrome because the femora and pelvis were found normal, thus the diagnosis of Fuhrmann syndrome was excluded [32]. For this reason, in the newest studies, these incidents were included as FATCO syndrome [11,33,34].

**Table 1:** Summary of clinical characteristics in previously reported cases of FATCO syndrome. FATCO: fibular aplasia, tibial campomelia, and oligosyndactyly

S. No.	References	Sex	Upper extremities	Lower extremities	Other malformations	Family history
1	Hecht and Scott (1981) [14]	Male	Bilateral hands absence	Left fibular aplasia, Left tibia aplasia, Bilateral foot absence	Cardiomegaly	Non-consanguineous couple, not mentioned family history of congenital or skeletal disorders
2	Hecht and Scott (1981) [14]	Female	Right hand absent, Left hand oligosyndactyly	Left fibular aplasia, Bilateral tibia shortening and campomelia, Right tibia with soft tissue dimpling, Bilateral foot oligosyndactyly	None	Non-consanguineous couple, not mentioned family history of congenital or skeletal disorders
3	Huber et al. (2003), Case 1 [32]	Male	Bilateral postaxial polydactyly	Bilateral fibular aplasia, Left tibia shortening, campomelia with skin dimpling, Bilateral oligodactyly, Bilateral clubfeet, Bilateral tarsal aplasia	Dislocation of hip	Non-consanguineous couple, not mentioned family history of congenital or skeletal disorders
4	Huber et al. (2003), Case 2 [32]	Male	Right hand oligodactyly	Bilateral fibular aplasia, Bilateral tibia campomelia with skin dimpling, Bilateral oligodactyly	Retrognathia, Ear lobule crease	Non-consanguineous couple, not mentioned family history of congenital or skeletal disorders
5	Cuillier et al. (2004) [30]	Not mentioned	Normal	Right fibula aplasia, Right tibia hypoplasia, Right foot oligodactyly, Right club-foot	None	Not mentioned affinity in couple, not mentioned family history of skeletal disorders
6	Courtens et al. (2005) [20]	Male	Left hand syndactyly	Right fibula aplasia, Right tibia shortening and campomelia with soft tissue dimpling, Right foot oligodactyly, Left foot dysmorphia	Mild micrognathia	Non-consanguineous couple, not mentioned family history of congenital or skeletal disorders
7	Monteagudo and Timor-Tritsch (2006) [31]	Female	Normal	Right fibula aplasia, Right tibia campomelia, Right foot oligosyndactyly, Right club-foot	None	Not mentioned affinity in couple, not mentioned family history of skeletal disorders
8	Kitaoka et al. (2009) [12]	Male	Right hand oligosyndactyly	Right fibula hypoplasia, Left fibula aplasia, Left tibia shortening with campomelia and soft tissue dimpling, Bilateral foot oligodactyly	Left cleft lip and cleft palate	Non-consanguineous couple, not mentioned family history of congenital or skeletal disorders
9	Karaman and Kahveci (2010)[34]	Male	Normal	Left fibula aplasia, Left tibia shortening with campomelia and soft tissue dimpling, Left oligosyndactyly	Not mentioned	Not mentioned affinity in couple, not mentioned family history of skeletal disorders
10	Vyskocil et al. (2011) [35]	Male	Normal	Left fibula aplasia, Left tibia shortening campomelia and skin dimpling, Left foot oligodactyly and calcaneous nucleous hypoplasia	None	Not mentioned affinity in couple, no family history of congenital disorders
11	Ekbote and Danda (2012) [37]	Male	Normal	Left fibula aplasia, Left tibia campomelia and soft tissue dimpling, Left oligosyndactyly	Klinefelter syndrome, Micropenis	Non-consanguineous couple, not mentioned family history of congenital or skeletal disorders
12	Bieganski et al.(2012), Case 1 [25]	Female	Bilateral oligosyndactyly	Bilateral fibular aplasia, Bilateral tibia camptomelia with skin dimpling, Bilateral oligodactyly, Bilateral tarso-calcaneal coalition and hypoplastic central ray, Bilateral equinous hindfoot, Bilateral tarsal coalition	Isolated membranous ventricular septal defect closed spontaneously	Non-consanguineous couple, not mentioned family history of congenital or skeletal disorders
13	Bieganski et al. (2012), Case 2 [25]	Male	Bilateral oligosyndactyly, Bilateral U-shaped hand,	Right fibular aplasia, Left fibular hypoplasia, Bilateral tibia campomelia with skin dimpling, Bilateral oligodactyly, Right plano-valgus foot, Left foot with talipes equinovarus	None	Non-consanguineous couple, family history unremarkable. Mother with bilateral partial skin foot syndactyly
14	Bieganski et al. (2012), Case 3 [25]	Male	Normal	Bilateral fibular aplasia, Bilateral tibia shortening and campomelia with skin dimpling, Bilateral oligosyndactyly	Not mentioned	Non-consanguineous couple, family history unremarkable
15	Sezer et al. (2014) [33]	Male	Right hand oligodactyly, Left hand oligosyndactyly	Bilateral fibular aplasia, Bilateral tibia shortening with camptomelia and skin dimpling, Bilateral oligosyndactyly, Bilateral club foot	None	Non-consanguineous couple, family history unremarkable
16	Goyal et al. (2014) [27]	Male	Normal	Right fibula hypoplasia, Right tibia campomelia with skin dimpling, Right foot oligosyndactyly, Right talus hypoplasia	Not mentioned	Non-consanguineous couple, family history unremarkable
17	Bastaki et al. (2014) [23]	Male	Normal	Bilateral fibular aplasia, Bilateral tibia camptomelia with skin dimpling, Bilateral oligosyndactyly,	None	Non-consanguineous couple, not mentioned family history of congenital or skeletal disorders
18	Smets et al. (2016) [38]	Female	Normal	Left fibular hypoplasia, Left tibia shortening with campomelia and soft tissue dimpling, Bilateral oligosyndactyly	None	Not mentioned affinity in couple, no relevant family history
19	D’Amato Gutiérrez and Palacio Díaz (2016) [39]	Male	Normal	Right fibular aplasia, Right tibia shortening and campomelia, Right foot oligodactyly	None	Not mentioned affinity in couple, not mentioned family history of skeletal disorders
20	Hazan et al. (2016) [40]	Male	Normal	Bilateral fibular aplasia, Bilateral camptomelia, Bilateral oligosyndactyly	None	Not mentioned affinity in couple, not mentioned family history of skeletal disorders
21	Nogueira et al. (2016), Case 1 [41]	Male	Ulnar deviation of the hands	Right fibular aplasia, Right tibia shortening and campomelia, Bilateral oligosyndactyly, Bilateral Rocker-Bottom foot	Intra-orbital grooves, Micrognathia, Bifurcated cardiac apex due to the left ventricle	Non-consanguineous couple, no family history of skeletal dysplasia
22	Nogueira et al. (2016), Case 2 [41]	Male	Normal	Left fibular aplasia, Left tibia campomelia with soft tissue dimpling, Bilateral oligosyndactyly, Bilateral clubfoot	Big phallus	Not mentioned affinity in couple, no family history of skeletal dysplasia
23	Nogueira et al. (2016), Case 3 [41]	Female	Normal	Right fibular aplasia, Right tibia campomelia with soft tissue dimpling, Bilateral oligosyndactyly	Micrognathia, Esophageal atresia with tracheal fistula	Not mentioned affinity in couple, no family history of skeletal dysplasia
24	Nogueira et al. (2016), Case 4 [41]	Male	Arachnodactyly	Left fibular aplasia, Left tibia campomelia, Left foot oligosyndactyly, Left tarsal bones hypoplasia	None	Non-consanguineous couple, no family history of skeletal dysplasia
25	Abdalla and El- Beheiry (2017) [22]	Female	Normal	Bilateral fibular aplasia, Bilateral tibia camptomelia with skin dimpling, Bilateral femoral angulation, Right foot oligodactyly, Left foot oligosyndactyly, Left foot aplasia of calcaneus, talus and cuboid bones, Left split- foot malformation	None	Non-consanguineous couple, no family history of congenital disorders
26	Amhad et al. (2017) [42]	Male	Not mentioned	Right fibular aplasia, Right tibia campomelia, Right foot oligodactyly, Right talus aplasia	None	Non-consanguineous couple, no family history of congenital disorders
27	Petricevic et al. (2017) [43]	Male	Right hand syndactyly	Bilateral fibular aplasia, Bilateral tibia camptomelia with soft tissue dimpling, Bilateral oligosyndactyly	None	Not mentioned affinity in couple, not mentioned family history of skeletal disorders
28	Önder Yılmaz et al. (2018) [36]	Female	Right hand with hypoplasia of fourth metatarsal bone, Left hand with duplication of fourth finger’s distal phalanx	Left fibular aplasia, Left tibia campomelia, Left foot oligodactyly, Right foot with duplication of the third metatarsus	None	Non-consanguineous couple, no family history of congenital or skeletal disorders
29	Guevara Zárate et al. (2018) [44]	Male	Not mentioned	Left fibular aplasia, Left tibia shortening and campomelia, Left foot oligodactyly	Not mentioned	Not mentioned affinity in couple, not mentioned family history of skeletal disorders
30	Isik et al (2019)[24]	Female	Left hand oligosyndactyly	Right fibular aplasia, Right tibia campomelia, Right foot oligodactyly	Ventricular dilatation and hydrocephaly	Non-consanguineous couple, not mentioned family history of congenital or skeletal disorders
31	Marinho et al. (2020), Case 1 [1]	Male	Normal	Right fibular aplasia, Right tibia shortening and campomelia. Bilateral feet oligosyndactyly, Right clubfoot	Micrognathia	Not mentioned affinity in couple, no family history of skeletal disorders
32	Marinho et al (2020), Case 2 [1]	Female	Normal	Right fibular aplasia, Right tibia campomelia with soft tissue dimpling, Right femur shortening, Bilateral oligosyndactyly	Micrognathia, Esophageal atresia with tracheal fistula	Not mentioned affinity in couple, no family history of skeletal disorders
33	Izadi and Salehnia (2020) [45]	Female	Normal	Right fibular aplasia, Right tibia shortening, Right foot oligosyndactyly, Right talus aplasia, Right calcaneus hypoplasia, Right split foot malformation	None	Consanguineous couple, no family history of congenital or skeletal disorders
34	Igoche and Umaru (2020) [46]	Female	Not mentioned	Left fibular aplasia, Left tibia shortening and campomelia, Left foot oligodactyly	Ventricular septal defect	Not mentioned affinity in couple, not mentioned family history of skeletal disorders
35	Kavipurapu et al. (2021), Case 1 [47]	Male	Left hand oligodactyly, Right hand syndactyly	Left fibular aplasia, Left tibia campomelia with skin dimpling	Spina bifida occulta	Consanguineous couple, family history was unremarkable
36	Kavipurapu et al. (2021), Case 2 [47]	Male	Normal	Right fibular aplasia, Right tibia campomelia with skin dimpling, Right femoral shortening and dysplasia, Right foot oligosyndactyly, Left foot oligodactyly, Right equino-valgus foot	Not mentioned	Non-consanguineous parents, family history was unremarkable
37	Mishra and Verma (2021) [28]	Male	Normal	Left fibula hypoplasia, Left tibia hypoplasia, Left foot oligosyndactyly, Left talus aplasia	None	Non-consanguineous couple, not mentioned family history of congenital or skeletal disorders
38	Yucel Celik et al. (2021) [21]	Male	Bilateral oligodactyly	Right fibular aplasia, Right tibia campomelia, Right foot oligodactyly, Bilateral clubfoot	Hyperechogenic bowel	Consanguineous couple, no family history of congenital or skeletal disorders
39	Matalon et al. (2022), Case 1 [15]	Male	Right hand symbrachydactyly	Bilateral fibular aplasia, Left foot syndactyly	None	Unknown
40	Matalon et al. (2022), Case 2 [15]	Female	Left hand symbrachydactyly	Bilateral fibular aplasia, Bilateral tibia shortening, Bilateral split foot malformation	None	Unknown
41	Matalon et al. (2022), Case 3 [15]	Male	Right hand oligosyndactyly, Left hand syndactyly	Right fibular aplasia, Right tibia shortening and slight campomelia, Bilateral oligosyndactyly	None	Not mentioned affinity in couple, not mentioned family history of skeletal disorders
42	Georgeos and Elgzzar (2022) [2]	Male	Right hand oligodactyly	Right fibula hemimelia, Right tibia campomelia with skin dimpling, Right foot oligosyndactyly (split foot malformation)	Brachycephaly	Consanguineous couple with strong history of genetic diseases and congenital anomalies
43	Benli et al. (2022)[26]	Female	Normal	Left fibular aplasia, Left tibia campomelia, Left foot oligodactyly	None	Non-consanguineous parents, not mentioned family history of congenital or skeletal disorders
44	Hashmi et al. (2022) [29]	Male	Normal	Right fibular rudimentary, Right tibia shortening and campomelia, Right foot oligodactyly	None	Consanguineous couple, no family history of limb abnormalities
45	Georgesku et al. (2022) [48]	Not mentioned	Normal	Right fibular aplasia, Right tibia shortening and campomelia, Right foot oligodactyly	None	Not mentioned affinity in couple, not mentioned family history of skeletal disorders
46	Sifre-Ruiz et al. (2023) [11]	Female	Normal	Right fibular aplasia, Right tibia shortening and campomelia, Right genu valgum and hyperflexion, Right foot oligodactyly, Right split foot malformation, Right clubfoot	None	Non-consanguineous parents, not mentioned family history of congenital or skeletal disorders
47	Current study	Male	Left ulnae hypoplasia, Bilateral oligosyndactyly, Bilateral split hand malformation	Right fibular aplasia, Right tibia shortening and campomelia, Bilateral oligosyndactyly	None	Non-consanguineous couple, not mentioned family history of congenital or skeletal disorders

Among all the reported cases, a wide range of variability has been demonstrated [35,36]. However, common findings in FATCO syndrome include the tendency to affect the right side, the anterior bowing of the tibia, and the skin dimpling located in the distal third [21], as observed in our case.

Based on our literature search, almost half of FATCO cases have been recorded in Asia with Turkey and India with the majority of cases. Interestingly, cases in which parental consanguinity was declared were also observed in these countries (Table 1) [37-48]. Based on clinical and radiographical findings, we report here the first case with FATCO syndrome presented in Greece demonstrating the classic triad in lower extremities: fibular aplasia, tibial campomelia, and bilateral oligosyndactyly. Concurrently, the diagnosis of SHFLD was made with the clinical detection of ectrodactyly of upper limbs and the presence of left ulnae hypoplasia on X-ray imaging. 

To date, the majority of published FATCO syndrome cases provided clinical data from the postnatal period. Our study is one of the few that highlights the fetal phenotypic features, aiming to familiarize fetal physicians with this rare condition, as prenatal diagnosis and counseling could be essential in the management of these cases. Taking into account the above-mentioned cases, it seems that our case is the second FATCO case overlapping with another limb syndrome, the first being reported by Abdalla et al. [22], which in this instance involves the upper limbs.

According to the most recent study, the involvement of the upper limb in FATCO syndrome seems to be rare, as only a few cases were recorded with upper limb defects, exclusively located in the hands [11]. For that reason, the presence of exhibited unilateral ulnae hypoplasia, which is a finding that had never been associated with FATCO syndrome (Table 1), raised the suspicion in our case that two distinct abnormal entities concurrently exist. Based on previously reported cases, a male preponderance has been established in FATCO syndrome [2] and the sex ratio (male/female), including our case, is now approximately estimated at 2:1 (Table 1).

## Conclusions

In this report, we presented an extremely unusual case of a fetus with a phenotype of FATCO syndrome associated with another rare upper limb disorder, SHFLD. To the best of our knowledge and based on the literature search presented, this is the first case with both FATCO syndrome and SHFLD reported. Considering the high clinical and genetic heterogeneity of these conditions, the diagnosis often seems to be difficult. Our case may contribute to further understanding of these rare conditions.
